# Therapeutic Maintenance of Janus Kinase Inhibitors in Real Life for Rheumatoid Arthritis: Retrospective Study

**DOI:** 10.3390/jcm13164608

**Published:** 2024-08-07

**Authors:** Camille Farnos, Vincent Barbier, Marie Doussiere, Valentine Deprez, Yannis Hamidou, Pierre Antoine Bruy, Jean Marc Sobhy Danial, Vincent Goeb

**Affiliations:** 1 Department of Rheumatology, Amiens University Hospital, 80054 Amiens, France; 2 Department of Physical Medicine and Rehabilitation, Victor Pauchet Amiens Clinic, 80090 Amiens, France

**Keywords:** rheumatoid arthritis, JAK inhibitors, baricitinib, tofacitinib, upadacitinib, filgotinib persistence

## Abstract

**Background/Objectives:** Janus kinase inhibitors (JAKis) belong to a new class of targeted oral drugs that have been added to the therapeutic arsenal for rheumatoid arthritis (RA). The aim of this study was to evaluate the efficacy and safety profiles of these four available molecules (tofacitinib, baricitinib, filgotinib, and upadacitinib) in real life. **Methods:** A retrospective, single-center observational study including all patients treated with JAKis for RA from 1 October 2017 to 1 December 2023. We assessed the maintenance rate at 24 months, which is an indirect reflection of the clinical and biological safety and efficacy profiles. **Results:** The 76 patients in our study were thus treated for the first time with anti-JAK, including 55 patients with baricitinib (BAR), 9 patients with tofacitinib (TOF), 4 patients with upadacitinib (UPA), and 8 patients with filgotinib (FIL). The majority of our patients had BAR introduced as the first intention. The therapeutic maintenance at 2 years for all our patients was 50%. The average maintenance duration was 8.6 months and was similar in all the groups. Of the 76 patients included in this study treated with Baricitinib (72.3%), 38 (50%) discontinued their treatment after two years of follow-up. **Conclusions:** Although this retrospective study is subject to various biases, it shows that the persistence rates of the four JAKi molecules in daily practice did not differ significantly, thus confirming the long-term efficacy of these drugs.

## 1. Introduction

Rheumatoid arthritis (RA) is the most common chronic inflammatory rheumatic disease, with a prevalence estimated at 0.5% of the French population [1]. It predominantly affects joints and has a chronic progression. This disease leads to progression joint damage, resulting in functional loss and a decreased quality of life [2].

The therapeutic management of RA is pivotal in rheumatology research and has undergone significant advancements over recent decades. The repertoire of biological agents has expanded considerably, and a new therapeutic class, Janus kinase inhibitors (JAKis), has recently emerged [3].

Janus kinases (JAKs) are essential signaling enzymes in the pathways of various cell surface cytokine receptors, including those involved in inflammatory processes. JAKis reduce inflammation by modulating the intracellular activity of JAKs and disrupting the signaling of multiple pro-inflammatory cytokines.

In 2017, two molecules were approved for regular clinical use in France: baricitinib (BAR) and tofacitinib (TOF), which were followed in 2019 and 2020 by upadacitinib (UPA) and filgotinib (FIL). They can be prescribed as second-line therapies following the inadequate response to disease-modifying antirheumatic drugs (DMARDs) [4,5,6,7].

Data on the efficacies and safety profiles of these agents in current clinical practice are available; however, information regarding their long-term therapeutic maintenance is still limited. Concerns have also emerged about a possible increased risk of venous and arteria thromboembolisms and cancer with TOF in the ORAL Surveillance study [8]. Following the results of this study, the Pharmacovigilance Committee of the European Medicines Agency (EMA) updated its recommendations in February 2022 [9].

The primary objective of this study was to investigate the therapeutic maintenance at 24 months and the tolerability of anti-JAKs in real life with greater hindsight since their launch in 2017. Real-life implementation provides a good reflection of a treatment’s safety and tolerability [10]. The secondary aims were to identify the factors associated with treatment discontinuation, efficacy profile, and adverse event collection.

## 2. Materials and Methods

### 2.1. Patient Selection 

This observational, single-center, and retrospective study was conducted in our rheumatology department between 1 October 2017 and 1 December 2023 and it included all patients who benefited from the introduction of JAKis. It was a continuation of work carried out in 2021 at the AP University Hospital by Dr Deprez, who studied the maintenance and tolerability of BAR and TOF in rheumatoid arthritis patients prior to the marketing of other JAKis currently available for rheumatoid arthritis [11]. The decision regarding the treatment to be administered was left to the discretion of the prescribing physicians according to the recommendations of the French Rheumatology Society. 

All patients were informed about this study’s objectives and provided consent for the anonymous use of their data in accordance with the Declaration of Helsinki. The protocol was approved by the Ethics Committee of the Department of Clinical Research and Innovation (DRCI) of the AP University Hospital and a conformity declaration to a reference methodology was made to the National Commission for Computing and Civil Liberties (CNIL) (project identification code: PI2020_843_002).

The inclusion criteria for patients were as follows: aged 18 years or older; with a confirmed RA diagnosis according to the 2010 American College of Rheumatology/European Alliance of Associations for Rheumatology (ACR/EULAR) 2010 criteria [12]; the initiation of JAKis in the Rheumatology Department of AP from 1 October 2017 to 1 December 2023, in accordance with the recommendations of rheumatology societies and following the failure of other previous treatments; and with no objection to the use of their data. 

The present study did not include patients who expressed opposition to inclusion or who were under legal protection measures. 

### 2.2. Data Recorded 

The data were gathered as part of the standard clinical practice at follow-up visits three months after the start of this study, and then every six months. Furthermore, computerized medical records were utilized. For each patient, the following data were extracted from the medical file:

- Demographic features (age, sex, body mass index, history of pulmonary, digestive and urogenital infections, neoplastic history, smoking, and Charlson comorbidity index (Supplementary Tables S1 and S2) [13]).
- RA characteristics (duration of disease, positivity of rheumatoid factor (RF) and anticitrullinated protein antibodies (ACPAs), presence of extra-articular manifestation (such as rheumatoid nodule, ocular involvement, cardiac involvement, pulmonary involvement, biological involvement, renal involvement, and vascular involvement), presence of erosion, and prior treatments and concomitant treatments).
- Cardiovascular risk (CV) (age, systolic blood pressure (mmHg, total cholesterol (mmol/L), HDL (mmol/L)) with a SCORE2 risk calculation for patients aged 40–69 (with no history of cardiovascular disease or diabetes), which is predictive of the 10-year risk of lethal or non-lethal cardiovascular events. The SCORE2-OP risk was also calculated at the start of treatment for patients aged over 70 to predict the risk of cardiovascular events at 5 or 10 years [14,15]. The score was adapted to EULAR recommendations [16]. The CV risk was divided into four categories according to the SCORE2 and SCORE2-OP risk calculations and the patient’s personal history: low CV risk, moderate CV risk, high CV risk, and very high CV risk.
- Clinical signs and symptoms were assessed at baseline and 3, 6, 12, 18, and 24 months and included the following: number of nocturnal awakenings, duration of morning rusting, number of swollen joints (NSJ), number of tender joints (NTJ), pain visual analog scale (VAS), patient global health VAS (hVAS), disease activity score in 28 joints using the erythrocyte sedimentation rate (DAS-28 ESR), disease activity score in 28 joints using C-reactive protein (DAS-28 CRP), and secondary effects (infections, neoplasia, or cardiovascular events).
- Laboratory findings were recorded at initiation and 3, 6, 12, 18, and 24 months, including the following: complete blood count (CBC), sedimentation rate (ESR), C-reactive protein (CRP), liver function, creatinine, and MDRD clearance.

Data were collected by this study’s principal investigator. A code was assigned to each patient that was known only to the investigator for anonymization purposes.

### 2.3. Statistical Analysis

Statistical analyses were performed using SAS software version 9.4 (SAS Institute Inc, Cary, NC, USA). The baseline characteristics were compared using the chi^2^ test or Fisher’s exact test for qualitative variables (expressed as numbers and percentages) and univariate analysis of variance or the Kruskal–Wallis test for quantitative variables (expressed as means and standard deviations for normally distributed data, or medians, minimums, and maximums for non-normally distributed data). Missing data were not imputed. 

The retention rates were estimated using the Kaplan–Meier method at 6, 12, 18, and 24 months and presented as survival curves. We conducted a comparison of these curves using the log-rank test. Censored variables, which pertained to patients who continued their treatment beyond 2 years, were included in the analysis.

Predictive factors of treatment retention were investigated using a Cox logistic regression model. Statistical tests were performed with an alpha risk of 0.05, and a *p*-value < 0.05 was considered significant. The relationship between treatment discontinuation and baseline characteristics was evaluated using a univariate Cox proportional hazards model. The results were reported as hazard ratios (HRs) with 95% confidence intervals (CIs). A *p*-value < 0.05 was considered statistically significant.

## 3. Results

### 3.1. Patient Selection and Characteristics 

Among the 76 patients included, 16 received a second line of JAKis after the primary inefficacy or escape of a first-line treatment. Given this very small sample size, no statistical analysis could be performed, and only the first JAKi intake was considered in this study. The 76 patients in our study were thus treated for the first time with JAKis, including 55 patients with BAR at a dosage of 4 or 2 mg per day, 9 patients with TOF at a dosage of 5 mg twice daily, 4 patients with UPA at a dosage of 15 mg once daily, and 8 patients with FIL at a dosage of 200 or 100 mg once daily. The initial demographic characteristics of our population are presented in Table 1.

Regarding the initial characteristics of the population, there was no significant difference according to the molecules used. Regarding the characteristics of RA, there was a significant difference (*p* = 0.001) in the duration of RA, with a shorter duration in the BAR group at 12.2 years and TOF at 14.8 years compared with the FIL and UPA groups, with averages of 24.8 and 19 years, respectively. There was also a significant difference (*p* = 0.02) regarding extra-articular manifestations of RA, with a predominance in the TOF group at 44% (*n* = 4) compared with the BAR group at 9% (*n* = 5) and an absence in the other groups, considering the small sample size.

### 3.2. JAKI Persistence

**Figure 1:**
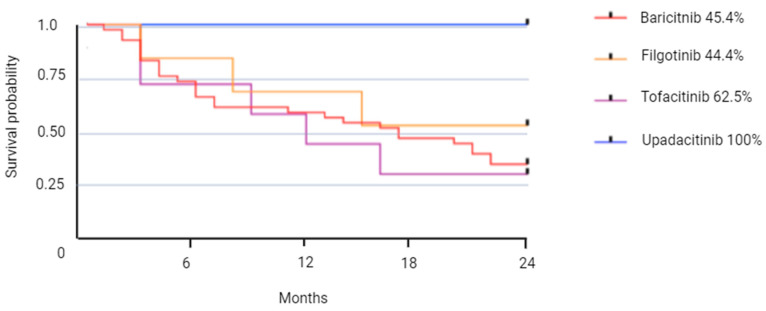
Kaplan–Meier estimations of the probability of Janus kinase inhibitors persistence at 2 years.

Therapeutic maintenance at 2 years for our entire cohort was 50% (*n* = 38), with 45.4% (*n* = 25) in the BAR group, 44.4% (*n* = 4) in the TOF group, 62.5% (*n* = 5) in the FIL group, and 100% (*n* = 4) in the UPA group (Table 2 and Figure 1). No significant difference in therapeutic maintenance was found between the four groups (*p* = 0.89). The average maintenance duration was 8.6 months and was similar in all groups. Of the 76 patients included in this study, 38 (50%) discontinued their treatment after two years of follow-up.

The reasons for the discontinuation of the JAKi treatments were as follows: primary inefficacy in nine patients (11.8%), escape in eight patients (10.5%), digestive intolerance in six patients (7.9%), biological intolerance in four patients (5.3%), cardiovascular causes in five patients (6.6%), desire for pregnancy in one patient (1.3%), and no discontinuation for neoplastic causes.

### 3.3. Predictive Factors for Therapeutic Maintenance

Our univariate analysis revealed that the Charlson score showed varied associations with the treatment maintenance. 

The Charlson score is a comorbidity indicator that quantifies the risk of mortality at 1 and 10 years. This score is calculated from 19 groups of comorbidities corresponding to a score ranging from 0 to 3 according to their severity. These scores are added together to calculate the Charlson score and predict the mortality risk. Our analysis ranged from a score of 1 to 6 units. A score of 1 unit is associated with low mortality at 1 and 10 years, which evolves in proportion to the increase in score. A high Charlson score is therefore associated with higher mortality. 

Some Charlson score levels were associated with an increased risk of treatment discontinuation (2 and 3 units), while others showed no significant association. These results did not allow us to predict whether this score was predictive of treatment maintenance, but these data remain an interesting avenue for further study (Table 3). 

No other demographic, clinical, or paraclinical characteristics were found to be associated with drug discontinuation.

### 3.4. Tolerance 

Regarding discontinuation due to infectious causes, there was a significant difference with a majority in the FIL group at 25% (*n* = 2) compared with 7.2% (*n* = 4) in the BAR group and no events in the TOF and UPA groups, considering the small sample sizes. The safety and efficacy of Filgotinib were evaluated in three phase III studies (FINCH 1, 2, 3), which did not reveal an increased infectious risk with this treatment [17].

The CV events recorded in our study were as follows: one case of uncontrolled hypertension, two pulmonary embolisms (one with deep vein thrombosis in the context of severe pulmonary infection with prolonged bed rest), unexplained lower limb lymphedema, and resting dyspnea without cardiac etiology found.

The infectious adverse effects documented in this study included exacerbations of chronic obstructive pulmonary disease, pneumonia, recurrent urinary tract infections, recurrent labial herpes, and a COVID infection requiring intensive care.

Biological abnormalities leading to treatment discontinuation were as follows: leukopenia (neutropenia and lymphopenia) with thrombocytopenia and worsening of severe chronic kidney disease.

### 3.5. Biological Surveillance

The safety of the JAKis was evaluated through the analysis of biological data, with a comparison of values at baseline with those at three and six months (Table 4). This study showed a slight decrease in hemoglobin (mean of −0.4 g/dL at 6 months). We also observed an increase in platelets (mean of +24,260 at 6 months) and a decrease in neutrophils (mean of 616 /mm^3^ at 6 months), but no difference in liver or kidney function or lipid profile.

### 3.6. Effectiveness Profile

**Figure 2:**
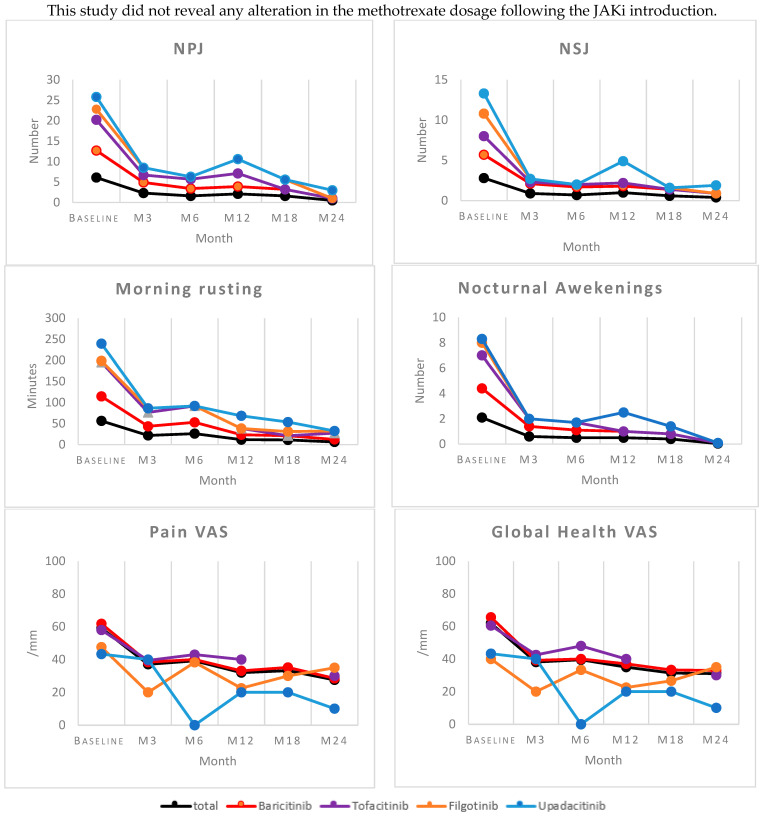
The effects of the intervention on morning stiffness, nocturnal awakenings, pain and global health VASs, and the numbers of tender and swollen joints were observed over a two-year period.

The evolution of clinical parameters is summarized in Figure 2.

For all molecules, we observed a reduction in clinical parameters used in daily practice after three months of treatment. This improvement persisted over time for the BAR, TOF, and FIL parameters, with continuous decreases in these values during the first six months of treatment, followed by relative stability between months 6 and 24. However, in the UPA group, there was an increase in the number of swollen and painful joints, the number of nocturnal awakenings, and the pain and disease VAS scores at 12 months, without any identified factors and with improvement at 18 and 24 months.

**Figure 3:**
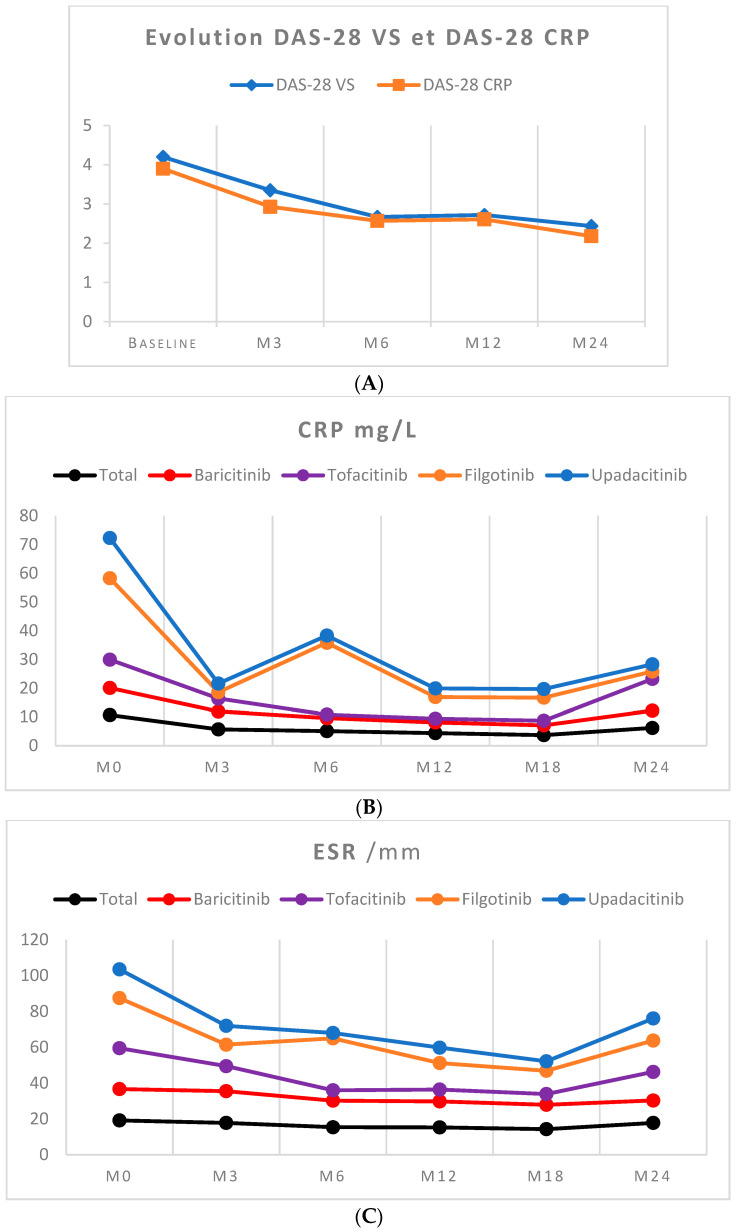
(**A**) Kinetic evolutions of DAS 28-ESR and DAS 28-CRP between initiation and 24 months. (**B**) Kinetic evolution CRP between initiation and 24 months. (**C**) Kinetic evolution ESR between initiation and 24 months.

After two years of treatment, there was a significant improvement in DAS28, with average decreases of 1.76 points for DAS28-ESR and 1.72 points for DAS28-CRP. These differences were statistically significant (Figure 3).

The inflammatory syndrome biomarkers decreased in all four groups. The patients treated with UPA showed a more rapid reduction in ESR (from 16 mm to 12.3 mm) and CRP (from 14 mg/L to 2.5 mg/L) at 3 months, with average decreases of 3.7 mm and 12.5 mg/L, respectively, between the baseline and month 6. These results should be considered cautiously due to our very small sample size for this molecule.

The mean ESR and CRP levels decreased by 1.2 points and 4.5 mg/L, respectively, for all treated patients. Specifically, there were decreases of 5 mm and 3.4 mg/L for the BAR group, a decrease of 6.8 mm and an increase of 1.2 mg/L for the TOF group, and decreases of 10.5 mm and 25.8 mg/L for the FIL group.

## 4. Discussion

The aim of this study was to investigate the real-life maintenance of JAKi agents in RA. The study of maintenance is a good reflection of the efficacy and tolerability of a treatment. The secondary objectives included identifying factors associated with treatment discontinuation, characterizing the therapeutic profile, and collating data regarding adverse events.

Our study evaluated the persistence of JAKis in 76 RA patients in routine practice in the rheumatology department of the AP University Hospital. The results demonstrated that 65.7% and 50% of patients, respectively, continued their treatment at 12 months and 24 months. Regarding the treatment tolerance, 7.9% presented with minor digestive intolerances (nausea, vomiting, abdominal pain), 5.3% with biological intolerances, and 6.6% presented with cardiovascular intolerances requiring treatment discontinuation. With the exception of one episode of hypertension with baricitinib and a predominance of infection with filgotinib, the other side effects were described in pre-marketing studies of anti-JAKs. It is notable that this small sample size included two cases of pulmonary embolism.

The demographic characteristics of our population were similar to those of patients in the RA-BEAM trial of baricitinib [18], but the patients were older and had a longer disease duration than those in the ORAL-STRATEGY study of tofacitinib [19]. Moreover, our patients generally had a lower disease activity at JAKi initiation [20]. This difference can be explained by the real-world setting of this study, where severe disease activity is unacceptable for the patient and practitioner, leading to short-course corticosteroid prescriptions during therapeutic changes, thus explaining the lower initial disease activity in our cohort.

Regarding the distribution of first-line JAKi treatment, the majority of our patients had baricitinib introduced as the first intention. This trend can be explained by greater experience since its commercialization in 2017 and the advantage of its single daily intake compared with tofacitinib, which was also marketed in 2017. Additionally, there may have been reluctance to prescribe tofacitinib following the European reevaluation of May 2019 of the A3921133 clinical study, which revealed an increased risk of severe venous thromboembolism at a dosage of 10 mg twice a day compared with anti-TNF [21].

The persistence of the drug at 24 months in our patients was comparable with other available treatments in RA, with no significant differences between the studied molecules. A 2022 study on a cohort of 205 patients found therapeutic maintenance rates at 2 years of 55.0%, 45.8%, and 62.4% in the infliximab, abatacept, and tocilizumab groups, respectively [22]. Another cohort study from the French REGATE registry showed maintenance rates of 61.3% for tocilizumab, 64.6% for rituximab, and 40% for abatacept at two years [23]. Concerning the comparison of the therapeutic maintenance of anti-JAK, few studies are available. We can cite the study by Martinez-Molina et al., which was published in 2024 and examined the efficacy and persistence of baricitinib, tofacitinib, filgotinib, and upadacitinib on a cohort of 189 patients for 12 months. The authors found a one-year maintenance rate of 51.4% [24]. In our study, we observed a higher one-year maintenance rate of 65.7%, which could be attributed to our smaller patient sample. Another study conducted at Amiens University Hospital found one-year maintenance rates of 67.6% for baricitinib and tofacitinib, which was comparable with our study [11].

Concerning the predictive factors for treatment retention, our study did not identify any significant predictors. The Charlson score showed varied associations with treatment retention, but we cannot draw definitive conclusions about its role. Further studies would be needed to investigate the Charlson score as a predictive factor for the retention of Jak inhibitors. A 2016 study highlighted poorer treatment retention in patients with a high Charlson score who were treated with anti-TNFα, abatacept, or tocilizumab [23]. Regarding the other factors studied, neither age, rheumatoid arthritis characteristics, cardiovascular risk, nor prior treatments showed any association with the treatment retention.

Regarding the efficacy profile of the treatment, our study showed a statistically significant improvement in the DAS 28 activity score at 2 years. We also demonstrated a decrease in clinical parameters used in daily practice as early as 3 months of use (NAD, NAG, VAS, RN, DM). Biologically, there was a significant reduction in the inflammatory markers after 6 months of treatment. Many studies had similar initial demographic criteria, but almost always showed higher disease activity (notably DAS 28, NAD, NAG, VAS pain, and disease) before initiation, which can explain a more significant differential in terms of the clinical response and efficacy. The real-world setting of our study might explain this difference, where high disease activity is unacceptable for both the patient and the practitioner, leading to the prescription of short-term corticosteroid therapy during treatment changes, thus explaining the lower initial disease activity in our cohort. 

Concerning the treatment tolerance, the two main causes of treatment discontinuation were primary inefficacy and escape, followed by adverse effects, which were comparable to the literature data [25]. Regarding the occurrence of venous thromboembolic events, it is notable that in our study, we recorded two cases of pulmonary embolism. However, thromboembolic risk has been described for tofacitinib and baricitinib at high doses, and recent meta-analyses are reassuring about the low risk of thromboembolic events at recommended doses in RA [26]. One of our patients was on baricitinib without misuse but had a major risk factor of severe pneumonia with prolonged bed rest. The second patient was on tofacitinib without misuse but had started treatment in 2019 before PRAC recommendations and had already had a pulmonary embolism episode. It is known that RA is intrinsically associated with a higher number of venous thromboembolic manifestations compared with the general population [27]. Concerning the occurrence of CV events, the main side effect observed was discontinuation due to uncontrolled hypertension under baricitinib, which is poorly described in the literature. Indeed, only 4.2% of 3464 patients in the baricitinib phase I and III studies experienced this effect [28]. Regarding infectious events, there was a predominance in the filgotinib group. The FINCH 4 study conducted in 2022, which analyzed filgotinib tolerance, showed a higher incidence of adverse effects in patients over 75 years old [29]. Another 2022 study on 3691 patients under filgotinib found a higher infection rate with the 200 mg daily dosage, while other adverse effects were comparable across dosages [30]. Complementary studies would be interesting to compare the infectious risk of filgotinib with other available JAKis.

The main strength of our study was the comparison of the four JAKi molecules available for RA with an extended follow-up period since their market introduction in 2017. Our long-term follow-up under real-world conditions allowed us to obtain an accurate representation of patients encountered in daily clinical practice. The real-world nature of our study was particularly interesting, as the majority of therapeutic studies had strict inclusion criteria for safety reasons, which largely excluded patients with multiple comorbidities or, conversely, patients with less severe clinical or biological expressions of inflammatory rheumatism. Nevertheless, these patients remained eligible for these treatments, which was a significant aspect of our real-world observation.

## 5. Conclusions

In contrast to randomized trials, which are considered the gold standard for establishing the effectiveness of treatments, real-world observational studies such as ours provide a better representation of the patients encountered in everyday practice, together with their long-term follow-up data. Therefore, they are crucial for assessing the effectiveness and safety of a medication. Our results showed comparable maintenance rates at 2 years among the four available JAK inhibitors in RA. They also confirmed their good efficacy in this indication. Primary inefficacy and treatment failure were the two main reasons for discontinuation. Regarding tolerance, despite the occurrence of two pulmonary embolisms in subjects with risk factors, there did not appear to be an increased cardiovascular or thromboembolic risk. We observed a higher rate of infectious episodes in the filgotinib group. Additionally, one case of hypertension under baricitinib required treatment discontinuation, highlighting the importance of regular cardiovascular monitoring in rheumatoid arthritis.

## Figures and Tables

**Table 1:** Baseline characteristics of the study population.

	Total n = 76	Baricitinib n = 55	Tofacitinib n = 9	Filgotinib n = 8	Upadacitinib n = 4	*p*-Value
Demographic characteristics						
Female sex, *n* (%)	63 (82.9)	45 (81.8)	7 (77.7)	7 (87.5)	4 (100)	0.82
Age (years), mean ± *SD*	56.8 (13.2)	55.0 (13.7)	64.6 (14.8)	58.6 (6.9)	60.0 (7.0)	0.21
BMI (kg/m^2^)	26.4	27.1	21.3	27.6	*	0.19
Systolic blood pressure (mmHg), mean ± *SD*	132 (14.5)	130 (13.2)	128 (10.0)	145 (19.0)	*	0.08
Personal medical history						
Pulmonary infection, *n* (%)	12 (15.8)	9 (16.3)	2 (22)	1 (12.5)	0 (0)	0.85
Digestive infection, *n* (%)	8 (10.5)	6 (10.9)	1 (11.1)	1 (12.5)	0 (0)	1.00
Urogenital infection, *n* (%)	7 (9.2)	4 (7.2)	1 (11.1)	1 (12.5)	1 (25)	0.76
Neoplasia, *n* (%)	8 (10.5)	5 (9.0)	3 (33.3)	0 (0)	0 (0)	0.12
History of MACEs and venous thromboembolism, *n* (%)	34 (44)	22 (40)	5 (55)	4 (50)	3 (75)	0.43
Anticoagulant treatment, *n* (%)	1 (1.3)	0 (0)	0 (0)	1 (12.5)	0 (0)	0.15
Anti-lipid treatment, *n* (%)	16 (21.1)	12 (21.8)	1 (11.1)	2 (25)	1 (25)	0.90
Smoking, *n* (%)	17 (22.4)	13 (23.6)	2 (22)	2 (25)	0 (0)	0.81
Charlson comorbidity index, mean	1.79	1.61	3.1	1.62	1.5	0.15
Cardiovascular risk **	*n* = 48	*n* = 35	*n* = 5	*n* = 7	*n* = 1	0.44
- Low risk	4 (8.3)	4 (11.4)	0 (0)	0 (0)	0 (0)	
- Moderate risk	17 (35.4)	13 (37.2)	2 (40)	2 (28.6)	0 (0)	
- High risk	19 (39.6)	12 (34.3)	3 (60)	4 (57.1)	0 (0)	
- Very high risk	8 (16.7)	6 (17.1)	0 (0)	1 (14.2)	1 (100)	
Characteristics of rheumatoid arthritis						
RF positive, *n* (%)	58 (76.3)	43 (78.1)	7 (77.7)	5 (62.5)	3 (75)	0.90
ACPA positive, *n* (%)	59 (77.6)	42 (76.3)	7 (77.7)	6 (75)	4 (100)	0.66
Disease duration, (years), mean ± *SD*	14.2 (10.6)	12.2	14.8	24.8	19	0.001
Erosion presence, *n* (%)	46 (60.5)	34 (61.8)	6 (66.6)	5 (62.5)	1 (25)	0.50
Extra-articular manifestations, *n* (%)	9 (11.8)	5 (9.0)	4 (44)	0 (0)	0 (0)	0.02
DAS 28-ESR, mean	4.2 (1.2)	4.8 (1.1)	4.1 (1.8)	3.5 (1.3)	3.7 (0.7)	0.57
DAS 28-CRP, mean	3.9 (1.1)	4.0 (1.1)	3.8 (1.3)	2.8 (0.9)	*	0.14
VAS pain, mean ± *SD*	59 (23.8)	61.6 (22.9)	58 (17.3)	47.6 (31.2)	44.3 (37.8)	0.59
VAS disease, mean ± *SD*	61 (22.9)	65.6 (20.0)	60.5 (23.1)	40 (29.1)	43.3 (37.8)	0.12
NPJ mean ± *SD*	6.1 (5.4)	6.5 (5.4)	7.7 (7.3)	2.6 (2.0)	3 (1.6)	0.24
NSJ mean ± *SD*	2.8 (3.2)	2.9 (3.4)	2.3 (2.7)	2.8 (2.5)	3.5 (2.6)	0.96
Number of nocturnal awakenings, mean ± *SD*	2.1 (2.1)	2.3 (2.2)	2.6 (1.9)	1 (1.7)	0.3 (0.5)	0.29
Morning stiffness (minutes) mean ± *SD*	56.4 (61)	58.8 (63)	81 (66.7)	3.3 (5.7)	40.6 (33.4)	0.10

*n*: number, *SD*: standard deviation, BMI: body mass index, MACEs: major adverse cardiac events, RF: rheumatoid factor, ACPA: anticitrullinated protein antibodies, DAS-28 ESR: disease activity score in 28 joints using the erythrocyte sedimentation rate, DAS-28 CRP: disease activity score in 28 joints using C-reactive protein, VAS: visual analog scale, NPJ: number of painful joints, NSJ: number swollen joints. * Due to the large number of missing data in patients treated with upadacitinib, statistical analyses could not be performed. ** Cardiovascular risk calculated for N = 48, 28 patients excluded from the calculation due to missing data..

**Table 2:** Two-year drug persistence of the four JAKi molecules.

		Total n = 76	Baricitinib n = 55	Tofacitinib n = 9	Filgotinib n = 8	Upadacitinib n = 4
Therapeutic maintenance, *n* (%)	6 months	55 (72.3)	38 (69.0)	6 (66.6)	7 (87.5)	4 (100.0)
	12 months	50 (65.7)	35 (63.6)	5 (55.5)	6 (66.6)	4 (100.0)
	18 months	43 (56.5)	30 (54.5)	4 (44.4)	5 (62.5)	4 (100.0)
	24 months	38 (50.0)	25(45.4)	4 (44.4)	5 (62.5)	4 (100.0)
Average retention time (months) [CI 95%]		8.6 [6.4–10.9]	8.6 [5.9–11.3]	8.6 [3.6–13.5]	8.6 [1.8–15.4]	
Number of stops, *n* (%)		38 (50.0)	30 (54.4)	5 (55.5)	3 (37.5)	0 (0)

**Table 3:** Factors associated with therapy discontinuation.

Parameter at M0	HR [CI 95%]	*p*-Value
Age at initiation	0.959 [0.835–1.101]	0.55
Disease duration at initiation	0.975 [0.870–1.093]	0.63
RF positive	1.493 [0.222–10.041]	0.68
ACPA positive	1.736 [0.370–8.150]	0.48
DAS 28-ESR at initiation	1.036 [0.438–2.448]	0.93
DAS 28-CRP at initiation	0.990 [0.381–2.574]	0.98
Low-risk CV	0.461 [0.011–19.394]	0.68
Moderate-risk CV	0.667 [0.089–4.996]	0.69
High-risk CV	0.368 [0.052–2.620]	0.31
Prior biotherapy		0.62
1–2	0.517 [0.056–4.766]	0.56
>=3	0.550 [0.153–1.969]	0.33
Charlson comorbidity index		0.17
1 unit	0.018 [0.000–1.831]	0.08
2 units	0.026 [0.001–0.723]	0.03
3 units	0.028 [0.002–0.470]	0.01
4 units	0.096 [0.005–1.744]	0.11
5 units	0.539 [0.046–6.355]	0.62
6 units	0.120 [0.005–2.807]	0.18

CV: cardiovascular risk, RF: rheumatoid factor, ACPA: anticitrullinated protein antibodies, DAS-28 ESR: disease activity score in 28 joints using the erythrocyte sedimentation rate, DAS-28 CRP: disease activity score in 28 joints using C-reactive protein.

**Table 4:** Biological evolution between baseline, 3, and 6 months.

Biological Evolution, Mean ± *SD*	Baseline	At 3 Months	At 6 Months
Hemoglobin (g/dL)	13.6 (1.34)	13.2 (1.2) *	13.2 (1.1) *
Platelets (10^3^/µL)	291.6 (103.6)	325.6 (94.7) *	315.8 (107.1) *
Leukocytes (10^3^/µL)	8.0 (3.0)	7.4 (2.1)	7.4 (1.9)
Polynuclear neutrophils (10^3^/µL)	4.9 (2.5)	4.3 (1.5)	4.4 (1.5)
Eosinophilic cells (/mm^3^)	207 (130)	161 (93) *	148 (84) *
Lymphocytes (/mm^3^)	2453 (1030)	2414 (1080)	2263 (1012)
Aspartate transaminase (UI/L)	21.9 (8.5)	26 (9) *	26 (9) *
Alanine transaminase (UI/L)	24.1 (15.1)	24 (14)	28 (15)
Creatinine (µmol/L)	68.8 (15.5)	69 (14)	69 (10)
Creatinine clearance (mL/min)	86.7 (23.2)	86 (15)	86 (14)
Total cholesterol ** (g/L)	2.08 (0.4)	2.0 (0.4)	2.1 (0.4)
Triglycerides ** (g/L)	1.2 (0.6)	1.2 (0.6)	1.2 (0.4)
LDL cholesterol ** (g/L)	1.1 (0.3)	1.1 (0.3)	1.2 (0.3)
HDL cholesterol ** (g/L)	0.6 (0.1)	0.6 (0.1)	0.6 (0.1)

*SD*: standard deviation, LDL: low-density lipoprotein, HDL: high-density lipoprotein. * Significant results with *p* < 0.05 with a post hoc Bonferroni analysis. ** Large number of missing data, with *n* = 24 at 3 months and *n* = 18 at 6 months. Missing data were not replaced.

## Data Availability

Data available on request due to ethical reasons.

## Supplementary Materials

The following supporting information can be downloaded from https://www.mdpi.com/article/10.3390/jcm13164608/s1. Table S1: Charlson score; Table S2: 10-year survival probability.
