# Supplementary material for: Therapeutic Maintenance of Janus Kinase Inhibitors in Real Life for Rheumatoid Arthritis: Retrospective Study
Source: J Clin Med. 2024 Aug 7;13(16):4608. doi: 10.3390/jcm13164608 (PMC11355502; doi:10.3390/jcm13164608)
Supplement: Supplementary file 1 [file jcm-13-04608-s001.zip › jcm-3121166-supplementary.pdf]

Table S1. Charlson score :

Condition	Score
Myocardial infarction	1
Congestive heart failure	1
Peripheral vascular disease	1
Cerebrovascular disease	1
Dementia	1
Chronic pulmonary disease	1
Connective tissue disease-rheumatic disease	1
Peptic ulcer disease	1
Mild liver disease	1
Diabetes without complications	1
Diabetes with complications	2
Paraplegia and Hemiplegia	2
Renal disease	2
Cancer	2
Moderate or severe liver disease	3
Metastatic carcinoma	6
AIDS/HIV	6

Table S2. 10-year survival probability

Total	10-year survival probability
0	99%
1	96%
2	90%
3	77%
4	53%
5	21%
§	2%
>6	0%
